# Multi-Objective Optimization of Thin-Walled Composite Axisymmetric Structures Using Neural Surrogate Models and Genetic Algorithms

**DOI:** 10.3390/ma16206794

**Published:** 2023-10-20

**Authors:** Bartosz Miller, Leonard Ziemiański

**Affiliations:** Faculty of Civil and Environmental Engineering and Architecture, Rzeszow University of Technology, Al. Powstancow Warszawy 12, 35-959 Rzeszow, Poland; bartosz.miller@prz.edu.pl

**Keywords:** shell, composite, optimization, surrogate model, genetic algorithms, artificial neural networks

## Abstract

Composite shells find diverse applications across industries due to their high strength-to-weight ratio and tailored properties. Optimizing parameters such as matrix-reinforcement ratio and orientation of the reinforcement is crucial for achieving the desired performance metrics. Stochastic optimization, specifically genetic algorithms, offer solutions, yet their computational intensity hinders widespread use. Surrogate models, employing neural networks, emerge as efficient alternatives by approximating objective functions and bypassing costly computations. This study investigates surrogate models in multi-objective optimization of composite shells. It incorporates deep neural networks to approximate relationships between input parameters and key metrics, enabling exploration of design possibilities. Incorporating mode shape identification enhances accuracy, especially in multi-criteria optimization. Employing network ensembles strengthens reliability by mitigating model weaknesses. Efficiency analysis assesses required computations, managing the trade-off between cost and accuracy. Considering complex input parameters and comparing against the Monte Carlo approach further demonstrates the methodology’s efficacy. This work showcases the successful integration of network ensembles employed as surrogate models and mode shape identification, enhancing multi-objective optimization in engineering applications. The approach’s efficiency in handling intricate designs and enhancing accuracy has broad implications for optimization methodologies.

## 1. Introduction

Composite shells find widespread applications across various industries, including aviation, machinery, and even construction [1,2]. These shells, known for their high strength-to-weight ratio and tailored material properties, have paved the way for innovative designs and improved performance in structural components. The efficacy of composite materials, however, is deeply intertwined with the meticulous selection of specific parameters, such as the matrix-to-reinforcement ratio and the orientation of the reinforcement, which play pivotal roles in shaping the mechanical behavior of these materials.

The optimization of these parameters has become a paramount pursuit, offering a means to attain desired characteristics and performance metrics [2,3,4]. The optimization process, whether aimed at achieving optimal dynamic responses, static stiffness under defined loads, critical buckling loads, material cost-effectiveness, or other engineering objectives, presents multifaceted challenges. Conventional gradient-based optimization techniques have proven efficacious in swiftly locating minima in many functions; however, their limitations are conspicuous when grappling with intricate, multimodal objective functions.

Amidst these complexities, stochastic optimization approaches emerge as promising alternatives, capable of approximating near-global optima in intricate, non-linear landscapes. Within the realm of stochastic optimization, evolutionary algorithms, drawing inspiration from biological processes, have garnered considerable interest from researchers. Among these, genetic algorithms stand out as prominent candidates for tackling optimization problems due to their ability to explore vast solution spaces effectively [5,6,7,8].

However, the wide-scale application of these methods is constrained by the computationally intensive nature of the optimization process. Conventional approaches necessitate a substantial number of objective function evaluations, particularly when the function values are computed using computationally intensive techniques such as Finite Element Method (FEM). This numerical burden prolongs the optimization process and exacerbates numerical instability issues.

The adoption of surrogate models emerges as a solution to alleviate these computational challenges [9,10]. Surrogate models, often based on artificial neural networks, offer an expedited means of approximating objective function values using a previously prepared dataset of patterns. Surrogate models enable faster and more efficient optimization procedures by circumventing the need for intricate numerical computations like FEM, significantly reducing the computational overhead.

Kalita et al. [11] conducted a comprehensive review of nearly 300 research articles on high-fidelity and metamodel-based optimization of composite laminates. This review emphasizes various metamodels and succinctly presents each research article’s methodologies and key outcomes, offering a valuable resource for future researchers and design engineers.

A global numerical approach for lightweight design optimization of laminated composite plates subjected to frequency constraints was presented in [12]. Their method utilizes an adaptive elitist differential evolution algorithm to solve the optimization problem with both integer and continuous variables, demonstrating its efficiency and reliability. Bargh et al. [13] applied the Particle Swarm Optimization (PSO) algorithm to optimize the lay-up design of symmetrically laminated composite plates for maximizing the fundamental frequency. The efficiency of the PSO algorithm was compared with the simple genetic algorithm, and the method’s effectiveness was validated against the existing literature results. In another study by Vo Duy et al. [14], the authors focused on multi-objective optimization problems of laminated composite beam structures. They aimed to minimize the beam’s weight and maximize its natural frequency. The study employed the Nondominated Sorting Genetic Algorithm II (NSGA-II) to tackle the optimization problem, showcasing the approach’s effectiveness for problems with both discrete and continuous design variables.

Tanaka [15] introduced a multi-objective optimization method for variable-thickness carbon fiber placement in composite laminates. The method aimed to achieve high strength and low weight by optimizing fiber orientation and thickness distribution using the Christensen fracture criterion and mean curvature as objective functions. In [16], the simultaneous optimization of stiffness and buckling load of composite laminate plates with curvilinear fiber paths has been tackled. This approach integrated surrogate modeling into an evolutionary algorithm, resulting in efficient optimization that simultaneously improved stiffness and buckling load over quasi-isotropic laminates.

Lee and Lin [17] presented a regression equation-based response surface approach to estimate the behavior of composite laminated structures, reducing the computational time required for optimization. The approach was validated with examples such as a marine propeller and a rotor wing, demonstrating both efficiency and accuracy. The same authors [18] enhanced a standard Genetic Algorithm (GA) by introducing local improvement and utilizing regression modeling for real calculation. The improved GA showed quicker convergence and significantly reduced calculation time. The approach’s efficacy was demonstrated through applications to a sandwich plate and composite propeller.

Drosopoulos et al. [19] proposed a multi-objective optimization study for the cost-effective design of nano-reinforced laminates. Their approach utilized the NSGA-II to optimize a hybrid laminate with conventional fibers and graphene nanoplatelets reinforcement. The optimization achieved enhanced fundamental frequency and reduced cost.

In [20], a global-local search strategy for the optimal design of laminated composite cylindrical shells with maximum fundamental frequency has been presented. The strategy employed the sequential permutation search algorithm for global optimization and the Ritz method for vibration analysis, offering a comprehensive approach for cylindrical shell design optimization. Sayegh [21] introduced an alternative approach to multi-objective optimization for detailed building models using reduced sequences in both sequential and adaptive strategies. These methods efficiently reproduced the Pareto front with reduced computational time and errors, showcasing their potential for optimizing complex systems.

The results of previous studies conducted by Miller and Ziemiański on single- and multi-objective optimization, including aspects such as maximizing the fundamental natural frequency, broadening frequency-free bands, and maximizing critical buckling load, have been presented in [22,23,24,25,26]. The present work significantly extends the scope of the previous findings.

In this context, this paper delves into the utilization of surrogate models in multi-objective optimization of composite shells. The study explores the application of Deep Neural Networks (DNN, see [27,28,29]) trained on a dataset of patterns to approximate objective function values, subsequently aiding in optimizing complex systems with diverse performance metrics. The investigation considers various optimization scenarios, including maximizing fundamental natural frequencies, optimizing frequency bandwidths, and optimizing cost parameters [30]. Additionally, the impact of mode shape identification and network ensembles on the performance of the optimization process is explored.

Surrogate models in multi-objective optimization: Surrogate models approximate complex simulations’ behavior, providing an efficient alternative to the computationally expensive Finite Element Analysis (FEA). In this study, surrogate models are employed to approximate the relationship between input parameters and the fundamental natural frequency ($f_{1}$), or width of frequency bands around different frequencies free from the structure’s natural frequencies. This allows for the exploration of a broad range of design possibilities without the need for a large number of FEA simulations.

Mode shapes identification and optimization accuracy: To improve the accuracy of surrogate models, mode shape identification is introduced as a preprocessing step. By identifying and analyzing mode shapes, the precision of surrogate models is enhanced, particularly in optimization of $f_{1}$ and frequency bands. The results demonstrate that the incorporation of mode shapes identification significantly improves the optimization process, providing more reliable and accurate Pareto fronts.

Utilizing network ensembles: To mitigate potential weaknesses in individual surrogate models, network ensembles are employed. These ensembles consist of multiple unique surrogate models, and the final predictions are selected from the best-performing model in the ensemble. Using network ensembles mitigates the risk of suboptimal results and ensures greater robustness and reliability in the multi-objective optimization process.

Efficiency analysis: One of the key aspects of our study is the analysis of the number of FE calls required for the optimization process. The computational effort is comprehensively assessed across various scenarios, including different sizes of training sets for surrogate models. This analysis provides valuable insights into the trade-off between computational cost and optimization accuracy.

Complexity of input parameters: The complexity of real-world engineering challenges often entails a high number of input parameters. In this research, 17 input parameters are considered, including geometrical parameters, material properties, and lamination angles. The approach is designed to manage such complexities efficiently, enabling thorough design space exploration.

Comparison with the Monte Carlo approach: To evaluate the effectiveness of our proposed methodology, a comparison is made between results obtained from surrogate-based multi-objective optimization and those derived from the classical Monte Carlo (MC) approach. The comparison highlights the superiority of the approach in terms of convergence and accuracy in capturing the Pareto fronts.

Normalization of Pareto front indicators: In multi-objective optimization, comparing Pareto fronts acquired from different problems can be challenging due to variations in the scale and nature of the objectives. A normalization method for Pareto front indicators is proposed to address this challenge, enabling fair comparisons and better decision-making.

This study demonstrates the successful application of surrogate models, mode shape identification, and network ensembles in enhancing multi-objective optimization. By efficiently handling a high number of input parameters, this approach provides more accurate and reliable results. Incorporating mode shape identification significantly improves the accuracy of the optimization process. We believe that these findings will have broad implications for various engineering applications, contributing to the development of efficient and effective optimization methodologies.

## 2. Problem Formulation

### 2.1. Generalized Eigenproblem and Mode Shapes Identification

The current study deals with structural dynamics. If the dynamic characteristic of the analyzed structure is investigated, the so-called generalized eigenproblem should be studied. This problem establishes a connection between structural properties, such as stiffness and mass matrices, and natural frequencies and vibration modes:

(1) $$K\Phi =M\Phi \Omega^{2},$$

where Φ matrix consists of the mode shapes of vibration $\phi_{i}$ (arranged in the columns of the matrix Φ), and Ω is a diagonal matrix with natural frequencies $f_{i}=\frac{\omega_{i}}{2\pi }$ adequate to the eigenvectors $\phi_{i}$. Typically, the natural frequencies are sorted in ascending order:

(2) $$f_{1}<f_{2}<\ldots <f_{n}.$$

The first and smallest natural frequency of vibration $f_{1}$ is commonly referred to as the fundamental frequency, since it plays a crucial role in analyzing the structure’s dynamic behavior. However, in complex structures subjected to varying dynamic loads, the higher natural frequencies become equally important.

The study of the mode shapes of the axisymmetric structure reveals that they can be classified into several distinct families:

- Axial modes (Anm), where the circumferential wave form number *n* is n=0, and the longitudinal wave form number *m* takes on natural number values m=1,2,…. The predominant displacement direction is along the axis of the structure;
- Torsional modes (Tnm), with n=0 and m=1,2,…, where the displacements in each cross-section have a direction perpendicular to the radius of the cross-section, remaining within the cross-section plane;
- Bending modes (Bnm), with n=1 and m=1,2,…, in which points on the same cross-section of the structure exhibit very similar displacements, resembling the behavior of an inextensible one-dimensional bar;
- Circumferential modes (Cnm), with n=2,3,… and m=1,2,…, specific to axisymmetric structures, where displacements along the radius of a cross-section with alternating signs dominate, forming characteristic waves.

The natural frequencies are assigned to appropriate mode shape families here, based on an analytic analysis of the geometry of each mode shape. Instead of arranging them in ascending order of increasing frequency, they are ordered according to the mode shape family to which they belong:

(3) $$f_{C21},f_{C22},\ldots ,f_{C31},f_{C32},\ldots ,f_{B11},f_{B12},\ldots ,f_{T01},\ldots$$

The subscripts next to the mode shape family name indicate the number of circumferential (the first) or longitudinal waves (the second) in the considered mode shape. For example, $f_{C21}$ represents the natural frequency corresponding to the C21 mode shape (the second circumferential mode shape with the first longitudinal waveform).

In the present study, the authors use an analytical mode shapes recognition procedure by analyzing the main component of displacement of the mode shape point with the highest displacement magnitude (see [23]).

**Figure 1:**
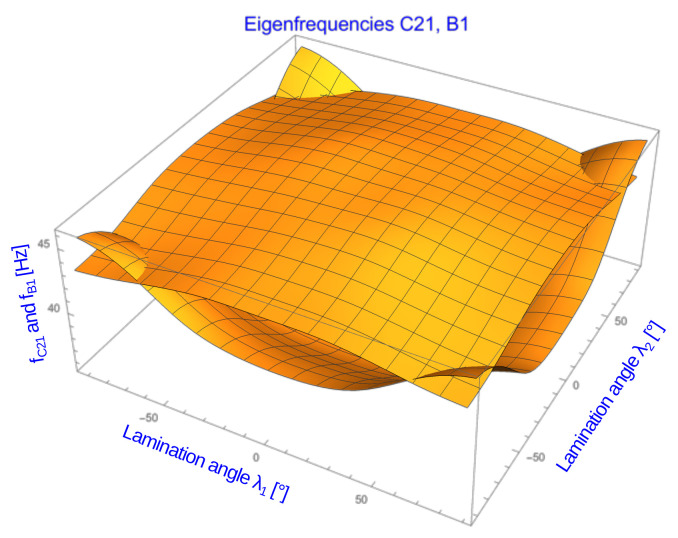
Mode shapes crossing for two varying lamination angles $\lambda_{1}$ and $\lambda_{2}$, frequencies corresponding to the first bending mode B1 and to the first circumferential mode C21.

The proposed approach allows for a more accurate estimation of the values of the eigenfrequencies. When the order of mode shapes belonging to different families (determined by the values of the corresponding natural frequencies) changes due to specific changes in the structure parameters, the dependence of the value of the frequency on the value of the changing parameter ceases to have a continuous derivative. This phenomenon is known as mode shapes crossing [23], and is illustrated in Figure 1 for a simple case with a simple model with two varying lamination angles $\lambda_{1}$ and $\lambda_{2}$ (lamination angles in two outer layers of a three-layer composite cylinder).

The points where the first derivative is non-continuous due to mode shapes crossing are nearly impossible to be precisely assessed using any surrogate model built based on the ascending order of natural frequencies. The advantages of using mode shape identification were presented in both single- and multi-objective optimization issues in [25,26], where selected dynamic parameters of the analyzed structure were optimized, along with optimizing its buckling behavior.

### 2.2. The Structure under Study

**Figure 2:**
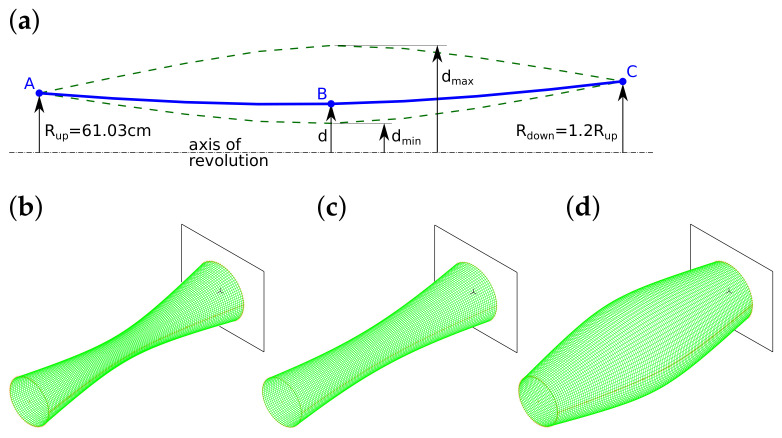
The structure under study: (**a**) hyperbola connecting points A, B, and C, (**b**) the most concave hyperboloid, (**c**) the hyperboloid with depth d=45 cm, (**d**) the most convex hyperboloid.

The investigated structure is a shell of revolution created by rotating a hyperbola connecting three points A, B, and C (see Figure 2a). Points A and C are fixed, while point B, situated in the middle of the hyperbola, moves along a line perpendicular to the axis of revolution. This motion results in shells with varying geometries (see the two dashed lines in Figure 2a and the corresponding extreme shapes of the hyperboloid in Figure 2b,d). Depending on the position of point B concerning the straight line connecting points A and C, the obtained shell of revolution can be a truncated cone, concave hyperboloid (if B is closer to the axis of revolution), or convex hyperboloid (if B is further away).

The radii of the end circles of the shell of revolution are fixed distances from the axis of revolution: $R_{up}$ and $R_{down}$. The distance of point B from the axis of revolution is referred to as the depth of the hyperboloid, denoted as *d* (see Figure 2a). The geometric parameters have the following values: the length of the hyperboloid (along the revolution axis) L=6.0 m, the upper radius $R_{up}=61.03$ cm, the lower radius $R_{down}=1.2R_{up}$, and the depth of the hyperboloid *d*, which varies between 30 cm (Figure 2b) and 110 cm (Figure 2d). The shell thickness is t=1.6 cm, divided into eight composite layers of equal thickness. The end of the analyzed shell, formed by the rotation of point C, is fixed, meaning all its displacements are constrained.

Each shell layer can be made of a different composite material, with different directions of the composite reinforcement fibers. The study considers three materials: Carbon Fiber Reinforced Polymer (CFRP), Glass Fiber Reinforced Polymer (GFRP), and a theoretical material (*t*FRP) whose properties and cost are calculated as the average of CFRP and GFRP. The introduction of *t*FRP aims to make the optimization problem, studied in the latter part of the article, more complex by considering more options than just two distinctly different materials. Table 1 provides a summary of all the applied materials properties (E${}_{\left(\cdot \right)}$: Young moduli, $\nu_{\left(\cdot \right)}$: Poission ratios, G${}_{\left(\cdot \right)}$: shear moduli of orthotropic material; *a* is the direction along the reinforcement fibers, *b* and *c* are the two directions perpendicular to the fiber direction named *a* here).

The investigated shell is described by seventeen varying parameters subjected to further optimization. These variable parameters are as follows:

- *d*, the depth of the structure, with 30cm≤d≤110cm;
- The material of each of the eight composite layers that constitute the structure shell, denoted by $\mu_{i}$, where i=1,2,⋯,8, and $\mu_{i}\in \left\{1,2,3\right\}$;
- The lamination angle of the eight composite layers, represented by $\lambda_{i}$, with i=1,2,⋯,8, and $-90^{\circ }\leq \lambda_{i}\leq +90^{\circ }$ (with a step of 5${}^{\circ }$).

The 17-element vector encompassing all the variable parameters of the investigated structure is denoted as p and defined as follows:

(4) $$\underset{17\times 1}{p}={\left\{d,\mu_{1},\mu_{2},\ldots ,\mu_{8},\lambda_{1},\lambda_{2},\ldots ,\lambda_{8}\right\}}^{\prime }.$$

### 2.3. Finite Element and Surrogate Models

The Finite Element (FE) model (see Figure 2b–d) comprises quadrilateral, multilayered shell 4-node MITC4 elements, employing the first-order shear theory. Each layer corresponds to one composite layer with possibly different material properties and lamination angles. The base size of the elements, denoted as *h*, is chosen to be nearly equal to h=5 cm. However, it slightly varies in the circumferential and longitudinal directions, as well as at different locations along the axis of the entire shell. All the FE calculations are performed in Adina code [31].

During the optimization process of the investigated structure’s dynamic properties, the number of dynamic property calculations corresponding to different values of the model parameters can reach several thousand. Utilizing the FE model leads to highly time-consuming numerical simulations. To overcome this issue, a neural network-based surrogate model is proposed.

The primary objective of the surrogate model is to promptly estimate, with satisfactory accuracy, the values of a selected number of natural frequencies corresponding to specific values of the investigated model parameters. For this purpose, DNN is employed as the surrogate model.

Various DNN-based surrogate models are considered, including models assessing 11 natural frequencies corresponding to 11 selected mode shapes or to the first 11 natural frequencies. The decision to choose 11 frequencies was based on the results of the authors’ previous studies [23]. Both preliminary analyses of the eigenfrequency spectrum for different values of the model parameters and test optimizations confirmed that expanding the set of identified frequencies was unnecessary. The mode shapes for which the natural frequencies are assessed by the DNN-based surrogate model are as follows: two bending modes (B11, B12), eight circumferential modes (C21, C22, C23, C31, C32, C33, C41, C42), and one torsional mode (T01). The surrogate model that assesses the first 11 natural frequencies is DNN${}^{nf}$, while the model evaluating the natural frequencies corresponding to the selected mode shapes is DNN${}^{id}$.

The task of the surrogate models is depicted as follows:

(5) $$\begin{array}{c} p\to \begin{array}{c} {\mathrm{DNN}}^{nf} \end{array}\to f^{nf},\\ p\to \begin{array}{c} {\mathrm{DNN}}^{id} \end{array}\to f^{id}, \end{array}$$

where $f^{nf}$ comprises the first 11 natural frequencies, and $f^{id}$ consists of the natural frequencies corresponding to the selected 11 mode shapes.

**Figure 3:**
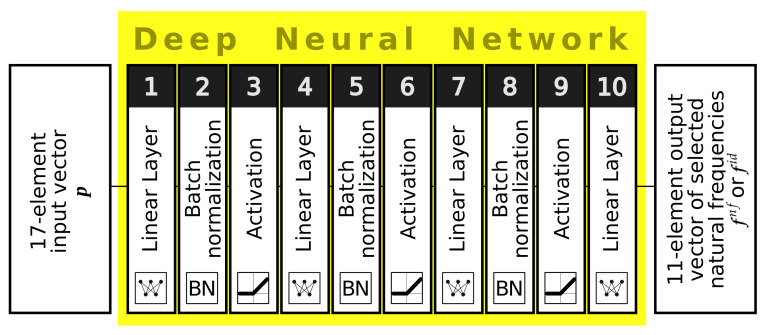
Single-network DNN${}_{S}$ surrogate model, each of the ten depicted layers is composed of 50 neurones. The only exception is the 11-neurones output layer (nb 10).

The surrogate models are applied either as single networks or in five-member teams (called network ensembles). Each neural network ensemble comprises five separate DNNs and returns the maximum value obtained among the ones calculated using the five single networks during the calculation of the objective function. A single-neural network surrogate model is denoted as DNN${}_{S}$ (a scheme of a single-network surrogate model is presented in Figure 3), while a surrogate model comprising five single networks is referred to as DNN${}_{E}$.

The validation of the optimization process outcomes is conducted through FEA. For the validation of outcomes stemming from five distinct approaches, each rooted in an individual surrogate model DNN${}_{S}$, an allocation of fivefold FE calls is necessitated. In a bid to circumvent this challenge, the proposition of network ensembles emerged, where each ensemble amalgamates five separate individual networks and identifies the maximal value from the set offered by these distinct networks. Ultimate testing is executed singularly for outcomes obtained via the ensemble surrogate models DNN${}_{E}$.

To create the networks building each surrogate model, supervised learning is applied. Teaching the network to reproduce the relationship between input and output data requires preparing a set of examples and presenting them to the networks. A crucial aspect of applying DNN-based surrogate models is to reduce the numerical effort (CPU time consumption) constantly. The overall CPU time consumed during the necessary number of FE calls, including generating examples for DNN-based model learning, must be significantly smaller than the CPU time consumption in case the surrogate model is not applied.

## 3. The Optimization Procedure

### 3.1. Optimization Flowchart

**Figure 4:**
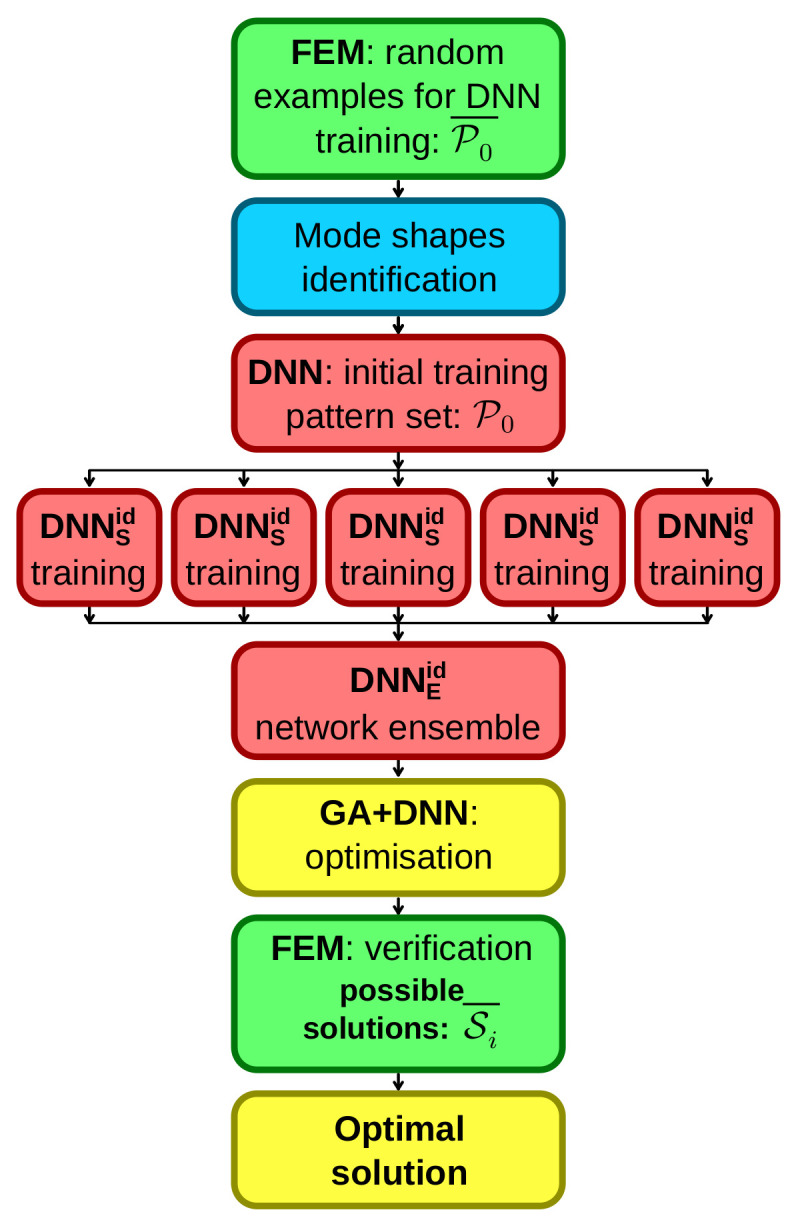
The flowchart of optimization; colors coding is as follows: green—FEM tasks, yellow—the optimization, blue—mode shapes identification, red—DNN tasks.

The optimization approach involving the DNN surrogate model is illustrated in the flowchart presented in Figure 4. The procedure commences with the construction of the DNN surrogate model, and it includes the following steps:

- Generate several FEM-based examples with random parameter vectors (set P);
- Identify the mode shapes and associate the natural frequencies with particular mode shape families (this step is omitted while creating the DNN${}^{fn}$ surrogate model);
- Train five independent DNN${}_{S}^{id}$ (or DNN${}_{S}^{nf}$) surrogate models to assess the values of natural frequencies, with an assumption of building a network ensemble DNN${}_{E}^{id}$ (or DNN${}_{E}^{nf}$).

### 3.2. Genetic Algorithms

The multi-objective optimization task is tackled using genetic algorithms, a metaheuristic global optimization algorithm inspired by nature. Among various metaheuristic algorithms tested (see [23]), GA has proven to deliver excellent results in optimization problems.

Genetic algorithms draw their inspiration from nature, specifically from a simplified representation of the genetic basis of evolution and natural selection in living organisms (see [32,33]). At each iteration step of the algorithm, a group of potential solutions (chromosomes) is considered. The best solutions, determined by evaluating the objective functions defining the optimization problem, are selected as parents for generating the next-generation solutions. The offspring solutions are created using specialized genetic operators, mainly involving pairing, crossover, and mutation. This iterative process of evaluating objective values for each possible solution, selecting the best solutions, and creating offspring continues until a satisfactory solution is achieved. Genetic algorithms belong to the group of zero-order algorithms, as they do not require the calculation of derivatives.

This study selects NSGA-II as the optimization algorithm, a well-established, efficient, and dependable algorithm [34,35]. The GA calculations are implemented using Matlab R2022b software [36].

### 3.3. Maximization of $f_{1}$ with Cost Minimization

Two optimization examples are investigated in this study. The first example involves a two-objective maximization of the fundamental natural frequency while minimizing the cost of the materials used. This optimization can be formulated as finding an optimal vector $p^{*}$ of model parameters p, which simultaneously minimizes the values of two objective functions $g_{f}\left(p\right)$ and $g_{c}\left(p\right)$:

(6) $$p^{*}=\underset{p\in P^{17}}{\arg\min}\left\{g_{f}\left(p\right),g_{c}\left(p\right)\right\},\;\;\;$$

where the objective functions are defined as follows:

(7) $$g_{f}\left(p\right)=-f_{1},$$

(8) $$g_{c}\left(p\right)=\mathrm{cost}\left(p\right).$$

The objective function $g_{f}\left(p\right)$ corresponds to the negative value of the fundamental natural frequency $f_{1}$. This means that the optimization aims to maximize $f_{1}$ in order to achieve a higher fundamental natural frequency.

On the other hand, the objective function $g_{c}\left(p\right)$ represents the cost of the structure, which is not calculated in any particular currency. The costs of the considered materials (see Table 1) are relative to each other. The final cost indicates how much one structure is more or less expensive than another, based on the relative material costs. This objective function aims to minimize the cost of the materials used in the structure.

The two-objective optimization aims to find a balance between maximizing the fundamental natural frequency and minimizing the material cost, leading to optimal solutions representing different trade-offs between these two objectives. The Pareto front obtained from this optimization contains non-dominated solutions, representing the best possible compromises between the competing objectives.

### 3.4. Maximization of Frequency Gaps with Costs Minimization

In the second optimization example, an optimal parameters’ vector $p^{*}$ is sought to minimize the values of two objective functions $g_{b}\left(p\right)$ and $g_{c}\left(p\right)$:

(9) $$p^{*}=\underset{p\in P^{17}}{\arg\min}\left\{g_{b}\left(p\right),g_{c}\left(p\right)\right\},\;\;\;$$

where the objective function $g_{b}\left(p\right)$ is defined in Equation (10):

(10) $$g_{b}\left(p\right)=-\min\left(F-f_{i}\left(p\right)\right).$$

Equation (10) defines the objective function such that it maximizes the width of the frequency band around an arbitrarily selected excitation frequency *F*, where F∈{50,60,70,80} Hz. In other words, the optimization aims to maximize the frequency range around the chosen excitation frequency free from structures’ natural frequencies. Please note that the value maximized is the distance from the center of the frequency band *F* to the nearest natural frequency $f_{i}$ of the analyzed structure. It is important to clarify that this distance is not literally the width of the frequency band itself—the actual width is at least twice as large, since it encompasses the space extending from the center of the band *F* in both directions (not only to the closest $f_{i}$). However, throughout the rest of this paper, the term bandwidth refers to the distance from the center of the frequency band *F* to the nearest natural frequency $f_{i}$.

The second objective function, $g_{c}\left(p\right)$, remains the same as in the previous optimization example, and represents the cost of the structure.

The optimization seeks to find the optimal values of the model parameters that result in the widest frequency band around the selected excitation frequency while minimizing the material cost of the structure. The Pareto front obtained from this optimization will provide various solutions representing different trade-offs between maximizing the frequency band and minimizing the material cost.

### 3.5. Multi-Objective Optimization Accuracy Assessment

In multi-criteria optimization, assessing the quality of the obtained approach is more complex than in single-criteria optimization. Instead of a single optimal solution, there is a set of solutions known as the Pareto front, where each point represents a non-dominated solution. A solution called A is non-dominated by solution B if the values of all objective functions obtained for A are not worse than those obtained for B, and at least one value is better.

In the case of two-criteria optimization considered here, the Pareto front forms a flat curve, with the axes of the coordinate system representing the two objective functions under consideration.

When comparing two Pareto fronts, the better one can be easily identified if none of its points is dominated by any point of the other front. However, determining the better one becomes difficult and sometimes even impossible if the fronts intersect, especially when comparing multiple fronts.

To aid in selecting a better front, performance indicators are employed [37]. From numerous available indicators, a carefully selected set of popular ones [38] is applied here to support the selection of the best option.

The Hypervolume indicator [39], denoted as $I_{H}$, provides information about the area covered by an investigated Pareto front and a reference point. When comparing several Pareto fronts, a common reference point should be selected. The higher the value of the Hypervolume indicator, the better. For direct comparison between two fronts, it can be redefined as the difference of two values, namely $I_{H}\left(A\right)-I_{H}\left(B\right)$. When one of the two Pareto fronts being compared is the true Pareto front (TPF, the best possible Pareto front sought during optimization), the indicator may be considered a unary $I_{H2}\left(A\right)=I_{H}\left(TPF\right)-I_{H}\left(A\right)$ indicator (smaller values of $I_{H2}\left(A\right)$ indicate that the considered front is closer to the TPF).

The Generational Distance indicator [40], denoted as $I_{GD}$, provides information on the distance of each point of the Pareto front to its closest point on TPF. A lower value of the Generational Distance indicator is desirable, as it signifies that the points on the front are closer to the true Pareto front.

The Epsilon ϵ-indicator [41], denoted as $I_{\epsilon }$, is a binary indicator that facilitates direct comparison between two Pareto fronts. The indicator $I_{\epsilon }\left(A,B\right)$ is the minimum scalar ϵ that scales Pareto front *B* in such a way that each point in ϵ·B is dominated by at least one point in *A*. If $I_{\epsilon }\left(A,B\right)<I_{\epsilon }\left(B,A\right)$, then Pareto front A may be considered better than Pareto front B. When the second Pareto front being compared is the true Pareto front, the indicator may be considered a unary $I_{\epsilon 1}\left(A\right)$ indicator.

In the tables presenting summaries of indicator values calculated for the obtained Pareto fronts, an arrow is employed to indicate the desired direction of change for a given indicator: ↑ signifies that a higher value of the indicator denotes a more favorable outcome, while ↓ signifies that a lower value corresponds to a more favorable result. This notation has been introduced for the convenience of the reader in interpreting the results.

## 4. The Results—Algorithmic Perspective

### 4.1. Introductory Remarks

The optimization process explored various configurations and combinations of surrogate models to find the most efficient and accurate approach. The following variations were considered:

1. Single vs. ensemble surrogate model: Two types of surrogate models were compared, namely a single neural network and an ensemble of neural networks. The single neural network, DNN${}_{S}$, is trained on a limited number of examples, while the ensemble of neural networks, DNN${}_{E}$, consists of five separate neural networks trained on the same examples (patterns). The ensemble approach aims to increase the robustness and generalization ability of the surrogate model;
2. Sorting or identifying natural frequencies: The natural frequencies obtained from the finite element simulations can be sorted in two ways, namely in ascending order or according to the corresponding vibration mode shape. The latter approach groups the natural frequencies based on the mode shapes they represent. The mode shapes provide valuable information about the structural behavior, which can be useful in certain optimization scenarios;
3. Necessary number of FE calls: The number of FE calls is pivotal from a computational burden standpoint. Estimating the minimum yet essential number of FE calls is crucial to optimize the computational load.

For each configuration, the optimization was performed with two objectives: minimizing the cost of materials and either maximizing the fundamental natural frequency ($f_{1}$) or maximizing the bandwidth around specific frequencies (50, 60, 70, or 80 Hz) free from any natural frequency.

Five training sets were created to assess the impact of the number of patterns necessary to train the surrogate models, each with a different number of elements. The surrogate models trained using their sets were denoted as V*x*, where *x* indicates the number of thousands of elements in the learning set (e.g., V05 corresponds to 500 elements, V1 to 1000 elements, V2 to 2000 elements, and so on).

To assess the quality of the Pareto fronts obtained during optimization, indicators requiring the use of TPF were used. Since there is no possibility to obtain the TPF (derive analytically or obtain by any other method) in the considered task, its role throughout the work is the envelope of all Pareto fronts obtained for the given problem by all methods described in the work.

The main goal of the analysis was to find the optimal combination of surrogate model configuration and learning set size that ensures accurate and efficient optimization results. The trade-off between computational effort and optimization performance was carefully evaluated, considering the quality of the obtained Pareto fronts and the convergence speed of the optimization process.

### 4.2. Surrogate Model Being a Single Network or an Ensemble of Five Networks

The first step of the analysis focused on comparing the results obtained from a single surrogate model, DNN${}_{S}^{id}$, with those obtained from network ensembles, DNN${}_{E}^{id}$. To conduct this comparison, five DNN${}_{S}^{id}$ surrogate models were created, each trained using the same set of examples. The optimization was performed with two objectives: maximizing the fundamental natural frequency ($f_{1}$) or maximizing the frequency bandwidth, both with cost minimization.

**Figure 5:**
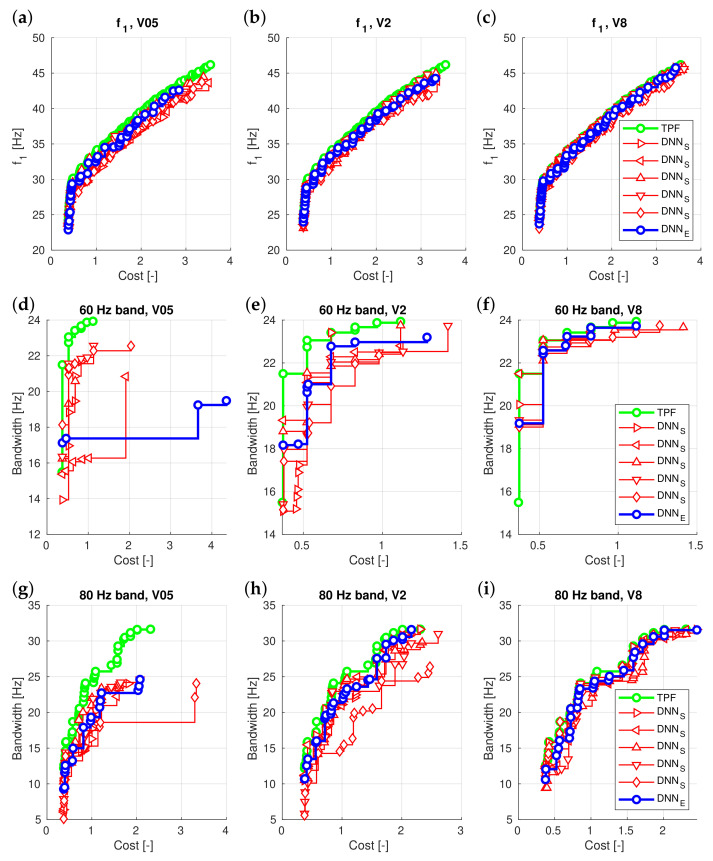
Surrogate models: DNN${}_{S}^{id}$ vs. DNN${}_{E}^{id}$ ensembles, (**a**–**c**): $f_{1}$ maximization, V05, V2 and V8 cases, respectively, (**d**–**f**): 60 Hz band maximization, V05, V2 and V8 cases, respectively, (**g**–**i**): 80 Hz band maximization, V05, V2 and V8 cases, respectively.

The results obtained from different DNN${}_{S}^{id}$ surrogate models and DNN${}_{E}^{id}$ ensembles are presented in Figure 5 (for the convenience of the reader, the data presented in these charts are in their original form, without scaling or normalization). For the Pareto fronts shown in Figure 5, various Pareto front indicators were calculated. Table 2 shows the values of indicators calculated for Pareto fronts obtained from different optimization approaches in the V05 learning set size case. Additionally, the table’s last row presents the data condensed, indicating the number of cases where DNN${}_{S}^{id}$ performed better than DNN${}_{E}^{id}$.

While it is straightforward to select the best-performing DNN${}_{S}^{id}$ surrogate model based on the results in Table 2, doing so requires running a series of FE verification for each individual surrogate model, significantly increasing the computational effort. To avoid this problem, DNN${}_{E}^{id}$ network ensembles were introduced.

With the network ensemble approach, the calculation of the objective function, $g_{f}\left(p\right)$ or $g_{b}\left(p\right)$, is modified. At first, five values are calculated using the five single surrogate models, and the final value is chosen as the best among them. The finite element verification is then performed only once, leading to significant computational savings.

The results presented in Table 3 demonstrate that the network ensembles effectively fulfill their intended task. Among the five models built on individual networks, the ensemble accurately selects the values to avoid the worst-case scenario. The average value from the data in the table does not exceed 1.6, with a median value of 1.

The findings indicate that using DNN${}_{E}^{id}$ ensembles successfully avoids the weakest model without the need to verify all individual models. While ensembles may not yield the absolute best results (see Figure 5), their purpose is to prevent obtaining the worst results and provide a reasonable trade-off between computational efficiency and optimization accuracy.

### 4.3. Surrogate Model Based on Identified Mode Shapes

In a previous study by the authors [23,24,25], it was demonstrated that incorporating mode shapes identification and analyzing natural frequencies with reference to the corresponding mode shapes leads to an improvement in the accuracy of the surrogate model and the overall optimization process. In the current research, this aspect was reevaluated, but this time, the focus was broadened to network ensembles, four different frequency bands, and fundamental natural frequency $f_{1}$. Moreover, multi-objective optimization was applied where besides the optimization of dynamic parameters also the cost of the structure has been taken into account.

**Figure 6:**
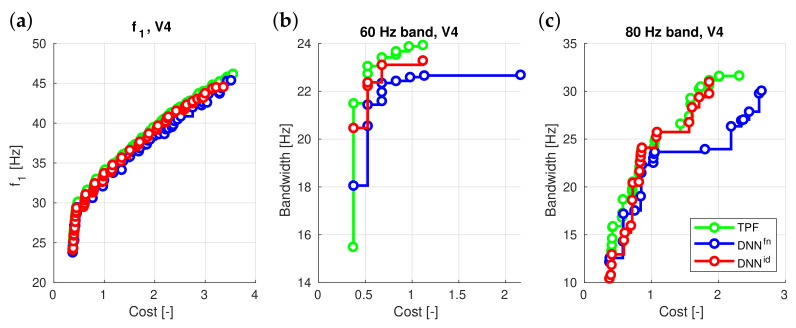
DNN${}_{E}^{id}$ vs. DNN${}_{E}^{fn}$ surrogate models: (**a**) $f_{1}$ maximization, V4 case, (**b**) 60 Hz band maximization, V4 case, (**c**) 80 Hz band maximization, V4 case.

Thus, the second issue examined was a comparison of the results obtained from two types of surrogate models: one working with identified mode shapes (DNN${}_{E}^{id}$) and the other with increasing values of natural frequencies without mode shape identification (DNN${}_{E}^{fn}$). The results are presented in Table 4 and in Figure 6.

Upon analyzing the results in the table, it becomes evident that surrogate models based on natural frequencies assigned to identified mode shapes of vibration exhibit clear advantages in most situations. This outcome highlights the importance of incorporating mode shape identification in the analysis of natural frequencies, as it leads to more accurate results and better optimization performance in the majority of cases.

Based on the results presented in the table, it is evident that mode shape identification plays an important role in enhancing the performance of the surrogate model in optimization.

### 4.4. The Influence of the Number of Patterns on the Optimization Accuracy

A wide range of simulations was conducted, covering five different optimization problems, each defined by two objective functions. In all cases, one common objective function was minimizing material costs. The second objective function allowed for the maximization of either:

- The fundamental natural frequency;
- The bandwidth around an arbitrarily chosen frequency, ensuring it was free from any natural frequencies of the shell.

Additionally, in each of the five described optimization problems, a surrogate model was applied, constructed from five neural networks (network ensemble), and trained with varying numbers of training samples (ranging from 500 to 8000 patterns). The number of FE calls used in each case was precisely calculated (including FE calls for verification of the obtained results), as one of the objectives of the proposed procedure was to minimize the number of FE calls.

**Figure 7:**
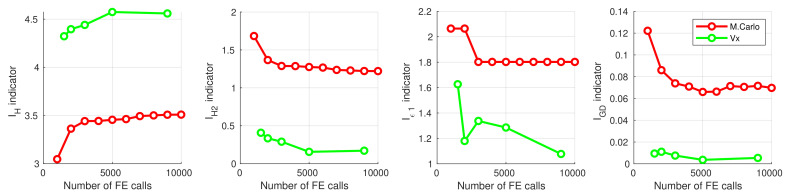
Pareto front indicators for different number of surrogate model learning patterns, $f_{1}$ maximization.

The results obtained from the optimization procedures using the neural surrogate models were compared with the results from the classical random Monte Carlo approach. Table 5 and Figure 7 present the results obtained in the maximization of $f_{1}$ with simultaneous cost minimization.

**Figure 8:**
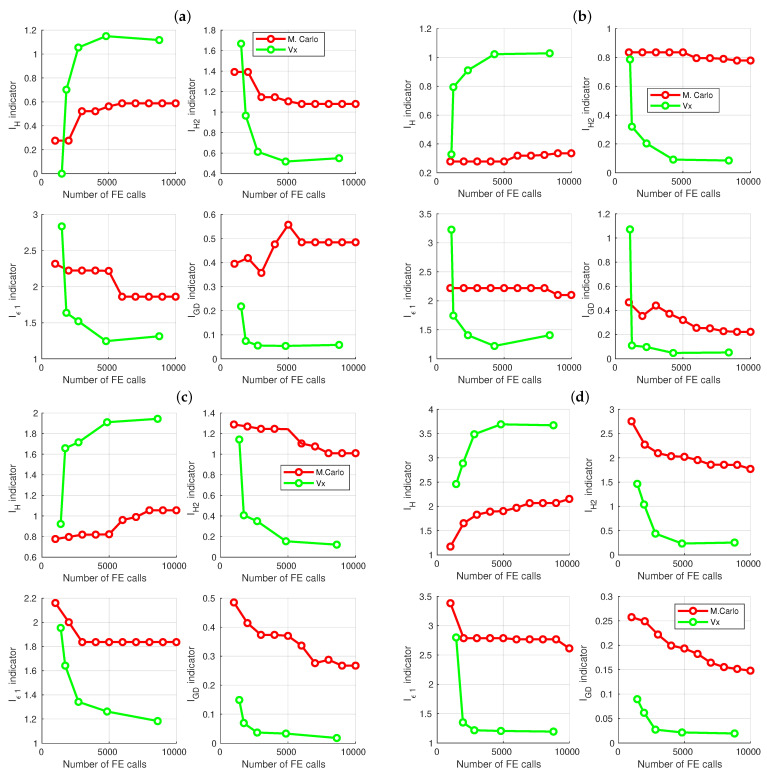
Pareto front indicators for different number of surrogate model learning patterns: (**a**) 50 Hz band maximization, (**b**) 60 Hz band maximization, (**c**) 70 Hz band maximization, (**d**) 80 Hz band maximization.

Table 6 and Figure 8 present the complete results of maximizing the width of four frequency bands, combined with cost minimization.

All the data presented in Table 5 and Table 6 and Figure 7 and Figure 8 indisputably demonstrate the significant advantage of using the GA for optimization over the random Monte Carlo method. This observation is consistent with expectations, but it is worth noting that a tenfold increase in the number of FE calls does not confer any advantage to the Monte Carlo method. The optimization approach proposed in this paper exhibits remarkable efficiency.

In all analyzed cases, a significant improvement in results was evident with an increase in the number of patterns used to train the surrogate models, up to a value of about 2000 patterns (case V2). Subsequently, the improvement in results became marginal, and in some instances, stagnation or even regression was observed. These findings suggest that the optimal number of patterns is 4000 (case V4)—for each of the analyzed cases, V4 consistently yielded either the best Pareto front indicator values or values close to the best. Further doubling the number of patterns to 8000 (V8) no longer resulted in significant improvement, and in certain cases, regression was observed.

**Figure 9:**
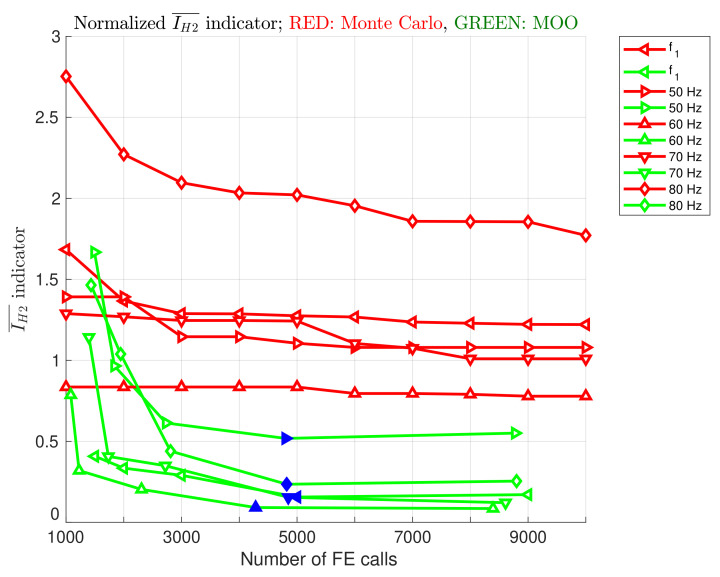
Normalized $\bar{I_{H2}}$, all optimization cases, the results obtained from the V4 optimization case are highlighted in blue.

The next figure, Figure 9, also shows an analysis of the quality of Pareto fronts obtained with different numbers of FE calls, but this time the normalized index $\bar{I_{H2}}$ was used to assess the quality of Pareto fronts. This approach made it possible to present the results obtained in different cases on a single chart. The value of $\bar{I_{H2}}$ indicator for a front named A is obtained according to the following formula:

(11) $$\bar{I_{H2}\left(A\right)}=\frac{I_{H2}\left(A\right)}{I_{H}\left(TPF\right)}=\frac{I_{H}\left(TPF\right)-I_{H}\left(A\right)}{I_{H}\left(TPF\right)}.$$

**Figure 10:**
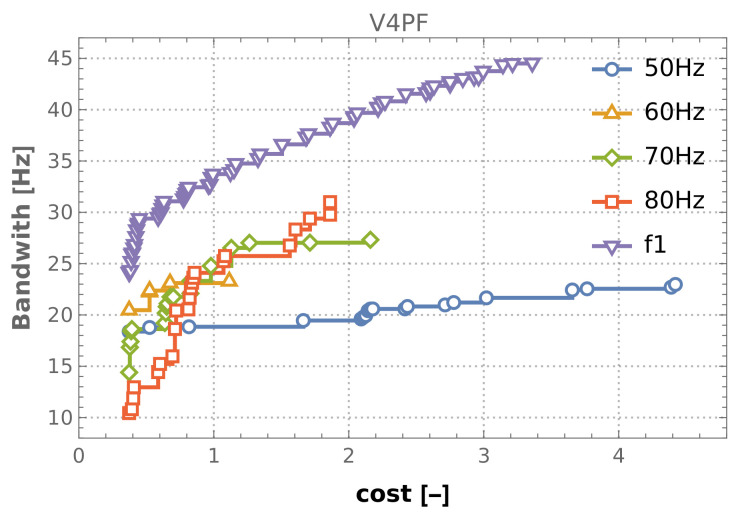
Pareto fronts, all optimization cases.

The results obtained from the V4 optimization case are highlighted in Figure 9 in blue. It is clear that no further improvement is observed in the V8 case. All Pareto fronts obtained from the V4 case, for all considered optimization cases (both $f_{1}$ maximization and four frequency bands width maximization), are collected in Figure 10. The maximal values of $f_{1}$ or bandwidths (coordinates of the right end of each Pareto front) obtained from the V4 case are collected in Table 7. It should be emphasized that the values given in the table cannot be regarded as the best solutions to optimization problems. They are only indications of what the largest values of $f_{1}$ and the widths of the intervals are found when performing optimization tasks.

## 5. The Results—Optimization Perspective

### 5.1. Depth d of the Structure

**Figure 11:**
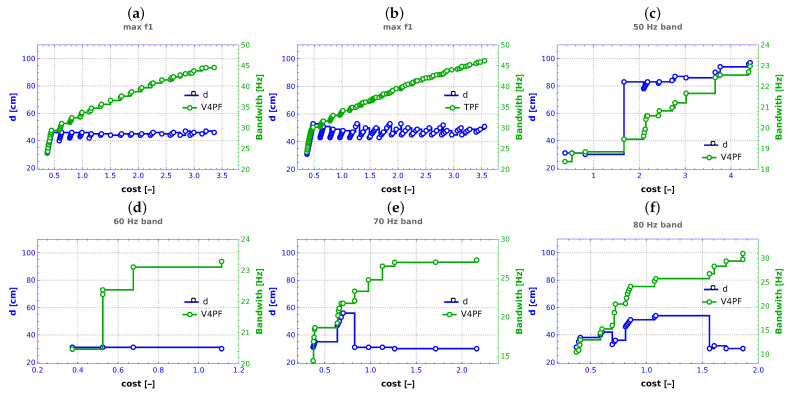
Final values of depth *d* in conjunction with associated Pareto front, (**a**) maximization of $f_{1}$, V4 case, (**b**) maximization of $f_{1}$, TPF, (**c**–**f**) maximization of bandwidth around frequencies 50–80 Hz, respectively.

The sole parameter that characterizes the geometry of the system under investigation is the depth, denoted as *d*. As illustrated in Figure 11, the optimization process yielded a range of values for the parameter *d*. For comparative purposes, the Pareto front acquired in the V4 case is shown, along with the corresponding *d* values.

The interpretation of the results is particularly straightforward when considering the maximization of $f_{1}$ (see Figure 11a). In this scenario, the parameter *d* consistently hovers around 45 cm. This shows that the optimal configuration for achieving the highest value of $f_{1}$ corresponds to a slightly concave hyperboloid shape (see Figure 2c). Notably, these same deductions can be drawn from an analysis of the TPF (see Figure 11b), not limited to the single front obtained through the earlier approach, denoted as V4. This emphasizes the robustness of the conclusions drawn from the considered optimization framework.

Since in the following cases, the TPF analysis leads to conclusions analogous to those that can be drawn from the analysis of a single V4 case, only the graphs for the V4 case are included.

For the scenario involving the optimization of bandwidth around 50 Hz (see Figure 11c), the parameter *d* follows a distinct trajectory. Initially, it assumes values slightly above its minimum considered value of 30 cm. However, it rapidly surges to values exceeding 80 cm, a range that corresponds to a configuration resembling a convex hyperboloid. Eventually, the parameter undergoes a slight incremental rise, surpassing 90 cm. When optimizing bandwidth around 60 Hz (see Figure 11d), the *d* parameter always reaches values very close to 30 cm. For the optimization scenarios centered around 70 Hz and 80 Hz bandwidths (see Figure 11e,f), the parameter *d* follows a distinct pattern. Initially, it adopts minimum values in proximity to 30 cm. Subsequently, it experiences a period during which it stabilizes at around 50 cm. However, for the 70 Hz cases, the parameter quickly reverts to its earlier values close to 30 cm. In contrast, this reversion occurs later in the context of the 80 Hz cases.

By scrutinizing the values of parameter *d* in conjunction with the associated Pareto fronts, a discernible pattern emerges. It becomes evident that significant distances between points along the Pareto front correlate with alterations in the material of one or multiple layers. Conversely, points situated in close proximity to one another on the front correspond to minor adjustments in parameters, such as *d*. Additionally, this relationship extends to lamination angles $\lambda_{i}$ as well. This observation elucidates the intricate interplay between the system’s geometry and material configuration in the context of multi-objective optimization.

### 5.2. Materials Selected in the Optimization Process

To facilitate the examination of the chosen materials throughout the optimization process, a parameter termed the material index (mi) is introduced. This index is defined by the equation:

(12) $$mi=\frac{1}{8}\sum_{i=1}^{8}\mu_{i}.$$

In the presented equation, the symbol $\mu_{i}$ corresponds to the material type linked with the *i*-th composite layer (where the total number of layers is eight). A material index of 3 signifies that all composite layers consist of material nb 3 (GFRP, as detailed in Table 1). Conversely, mi=1 indicates the utilization of the strongest and costliest CFRP for all layers. Notably, a material index of 2 indicates a balanced distribution, with an equal number of layers constructed from GFRP and CFRP, while the remaining layers are composed of *t*FRP.

The material index offers a succinct representation of the material configuration choices made during the optimization process, providing insights into the composition of the composite structure.

**Figure 12:**
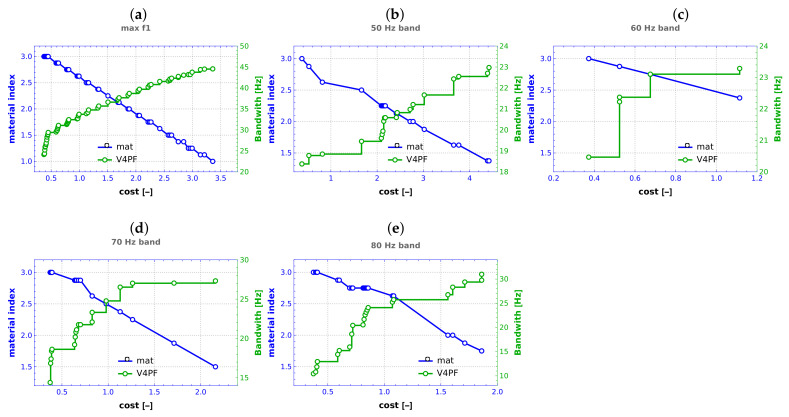
Final values of material index mi in conjunction with associated Pareto front obtained from V4 case, (**a**) maximization of $f_{1}$, (**b**–**e**) maximization of bandwidth around frequencies 50–80 Hz, respectively.

As anticipated in the context of multi-objective optimization, where one of the minimized objective functions is the cost function, there is a notable correlation between the increase in cost and the proportion of pricier materials (see Figure 12). Interestingly, however, this trend is observed exclusively in the task of maximizing $f_{1}$, where the Pareto front encompasses a wide spectrum of scenarios, spanning from the most economical to the most costly alternatives (see Figure 12a). In contrast, within the realm of bandwidth optimization, the outcomes tend to cluster around the cost range associated with more economical materials. For instance, in the scenario of bandwidth optimization around 50 Hz, the attained results exhibit a range of mi values extending from 3 (representing the most affordable option) to approximately 2.4, with the highest-cost solutions (characterized by mi values close to 1) not being favored at all in this context (see Figure 12c).

### 5.3. Lamination Angles

**Figure 13:**
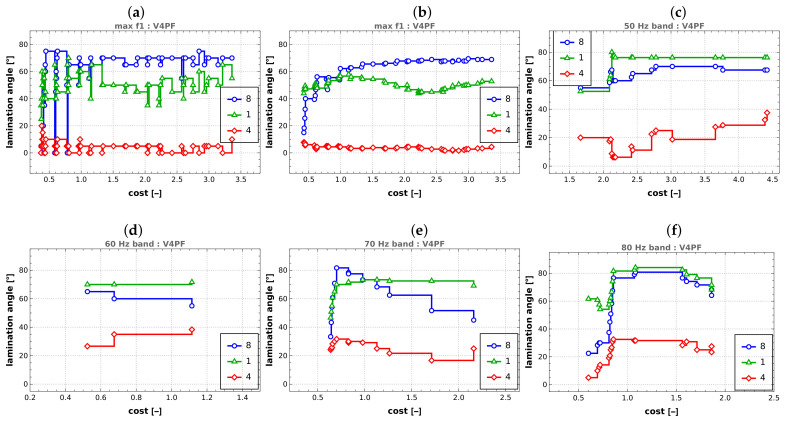
Lamination angles, (**a**) maximization of $f_{1}$, original values, (**b**) maximization of $f_{1}$, averaged values, (**c**–**f**) maximization of bandwidth around frequencies 50–80 Hz, respectively, averaged values.

The analysis of lamination angles (see Figure 13a) presents a more intricate challenge, and it does not readily yield overarching insights that can be applied across all the optimization scenarios under consideration. The chart in Figure 13a presents the values of lamination angles for three selected layers: inner (layer 1), middle (layer 4), and outer (layer 8). Each point on the chart corresponds to consecutive points on the Pareto front obtained in the V4 case. The results directly read from the Pareto front were averaged for several neighboring points to analyze trends and draw general conclusions regarding the obtained lamination angle values. The values of the resulting moving average are depicted in Figure 13b. Clear tendencies are visible, distinctly different for the inner/outer and middle layers. Lamination angles of outer and inner layers tend to approach approximately 70 degrees (outer layer) or 50 degrees (inner layer) with increasing cost (and concurrently increasing obtained $f_{1}$ value). Lamination angles of middle layers tend towards values close to zero.

Only averaged data are presented for tasks related to the maximization of four bands. In all cases, a phenomenon similar to the maximization of $f_{1}$ can be observed: lamination angles of the inner and outer layers tend to have significantly larger values than those of the middle layers.

## 6. Summary of Main Research Findings

The effectiveness of single neural network surrogate models (DNN${}_{S}$) was compared to network ensembles (DNN${}_{E}$), each consisting of five neural networks. The goal was to ascertain which approach provided more reliable results. Although individual DNN${}_{S}$ models showed varying performance, DNN${}_{E}$ ensembles proved effective in selecting the best outcome without the need for extensive verification (Table 3). The ensemble approach strikingly mitigated the risk of suboptimal results by combining the strengths of multiple networks.

The impact of mode shape identification on surrogate models was reevaluated, comparing identified mode shapes (DNN${}_{E}^{id}$) with sorted natural frequencies (DNN${}_{E}^{fn}$). Results consistently favored the mode shape-based approach (Table 4, Figure 6), underlining the importance of incorporating mode shape information to enhance surrogate model accuracy.

The influence of the number of training patterns on optimization efficiency was analyzed across five optimization problems. Surrogate models trained with approximately 4000 patterns (V4) showed optimal performance, with diminishing returns observed beyond this point. The proposed method vastly outperformed the Monte Carlo approach, affirming its computational efficiency and robustness.

The parameter *d*, representing the structure’s depth, played a pivotal role in the optimization process. Depending on the objective, such as maximizing the fundamental natural frequency ($f_{1}$), *d* consistently favored a certain value. Modeled as a slightly concave hyperboloid, this configuration ensured maximum $f_{1}$ (Figure 11).

The material index (mi) was introduced to characterize material composition choices. For $f_{1}$ maximization, a correlation between cost and material index was observed, ranging from cost-effective to high-cost scenarios. In contrast, bandwidth optimization resulted in solutions clustering around economical materials, highlighting the optimization’s economic efficiency (Figure 12).

The analysis of lamination angles revealed intricate trends. Lamination angles for inner and outer layers approached 70 and 50 degrees, respectively, with increasing cost, while middle layers tended towards near-zero angles. This nuanced behavior underscores the intricate interplay between material composition and geometric configuration.

In conclusion, the optimization process demonstrated the efficacy of ensemble surrogate models, the significance of mode shape identification, and the efficient trade-off between computational effort and optimization performance. The parameter *d*, material composition, and lamination angles intricately influenced optimization outcomes. The approach showcases potential for various engineering applications, offering a comprehensive framework for efficient and accurate optimization.

## 7. Final Remarks

This research addresses the challenges of multi-objective optimization with a high number of input parameters in engineering applications. Through the use of surrogate models, mode shape identification, and network ensembles, a novel, efficient approach has been introduced to tackle complex optimization problems. Several key contributions and avenues for future research are highlighted by these findings:

- The incorporation of surrogate models has proven to be a powerful technique for approximating the behavior of complex simulations;
- The mode shapes identification step has emerged as an important step in enhancing the precision of surrogate models;
- The utilization of network ensembles has significantly enhanced the robustness of the optimization process, while also substantially decreasing the required number of FE calls to validate the achieved optimization outcomes;
- The analysis of the number of finite element calls required for optimization has shed light on the trade-off between computational effort and optimization accuracy;
- The complexity of input parameters has been addressed by handling 17 variables encompassing geometrical parameters, material properties, and lamination angles;
- The comparison of the results with the classical Monte Carlo approach has solidified the superiority of the methodology.

The integration of surrogate models, mode shape identification, and network ensembles has proven to be a highly effective and efficient methodology. The findings are expected to inspire further exploration and advancements in this field, and the application of the approach in various real-world engineering challenges is eagerly anticipated.

## Figures and Tables

**Table 1:** Material properties of layered composites applied in the investigated model.

Material	μ	E${}_{a}$	E${}_{b}$	E${}_{c}$	$\nu_{\mathrm{ab}}$	$\nu_{\mathrm{ac}}$	$\nu_{\mathrm{bc}}$	G${}_{\mathrm{ab}}$	G${}_{\mathrm{ac}}$	G${}_{\mathrm{bc}}$	Density	Cost
**Name**	**[-]**	**[GPa]**	**[GPa]**	**[GPa]**	**[-]**	**[-]**	**[-]**	**[GPa]**	**[GPa]**	**[GPa]**	**[** $\mathrm{kg}/m^{3}$ **]**	**[-]**
CFRP	1	120	8	8	0.014	0.028	0.028	5	5	3	1536	10.20
*t*FRP	2	80	6	6	0.020	0.036	0.036	4	4	3	1428	5.78
GFRP	3	40	4	4	0.026	0.044	0.028	3	3	3	1320	1.36

**Table 2:** Surrogate models: DNN${}^{id}$ vs. DNN${}_{E}^{id}$ ensembles, $f_{1}$ maximization with V05 networks.

	$I_{H}$↑	$I_{H2}$↓	$I_{\epsilon 1}$↓	$I_{\mathrm{GD}}$↓
Ensemble DNN${}_{E}^{id}$	4.32	0.408	1.63	0.009
Single DNN${}_{S}^{id}$	4.04	0.691	1.59	0.023
Single DNN${}_{S}^{id}$	4.20	0.528	1.44	0.017
Single DNN${}_{S}^{id}$	4.40	0.326	1.30	0.012
Single DNN${}_{S}^{id}$	4.16	0.569	1.78	0.015
Single DNN${}_{S}^{id}$	4.10	0.634	1.43	0.021
The number of single models DNN${}_{S}^{id}$ better than the ensemble DNN${}_{E}^{id}$	1	1	4	0

**Table 3:** The number of single networks (DNN${}_{S}^{id}$) better than the ensemble (DNN${}_{E}^{id}$) results.

	V05				V1				V2				V4				V8			
$f_{1}$	1	1	4	0	1	1	0	2	1	1	3	2	1	1	2	0	2	2	3	2
50 Hz	3	3	4	3	0	0	0	0	0	0	4	1	1	1	1	1	2	2	1	3
60 Hz	4	4	5	5	4	4	5	2	0	0	1	1	0	0	0	0	1	1	3	2
70 Hz	1	1	1	3	1	1	4	1	2	2	1	1	0	0	0	1	3	3	3	3
80 Hz	2	2	0	3	2	2	0	0	0	0	2	0	0	0	2	1	2	2	2	2

**Table 4:** Surrogate models: DNN${}_{E}^{id}$ vs. DNN${}_{E}^{fn}$; V4 case.

V4	$I_{H}$↑	$I_{H2}$↓	$I_{\epsilon 1}$↓	$I_{\mathrm{GD}}$↓
$f_{1}$ maximization				
DNN${}_{E}^{id}$	4.57	0.156	1.29	0.004
DNN${}_{E}^{fn}$	4.41	0.318	1.14	0.011
50 Hz band				
DNN${}_{E}^{id}$	1.15	0.518	1.25	0.054
DNN${}_{E}^{fn}$	0.96	0.706	1.60	0.060
60 Hz band				
DNN${}_{E}^{id}$	1.02	0.091	1.22	0.048
DNN${}_{E}^{fn}$	0.88	0.232	1.43	0.129
70 Hz band				
DNN${}_{E}^{id}$	1.91	0.154	1.26	0.034
DNN${}_{E}^{fn}$	1.39	0.673	1.54	0.097
80 Hz band				
DNN${}_{E}^{id}$	3.69	0.235	1.21	0.022
DNN${}_{E}^{fn}$	2.88	1.048	1.50	0.075

**Table 5:** Pareto front indicators for different number of surrogate model learning patterns, $f_{1}$ maximization.

	FE Calls	$I_{H}$↑	$I_{H2}$↓	$I_{\epsilon 1}$↓	$I_{\mathrm{GD}}$↓
MC	1000	3.05	1.684	2.06	0.122
MC	2000	3.36	1.367	2.06	0.086
MC	3000	3.44	1.288	1.80	0.074
MC	4000	3.44	1.287	1.80	0.071
MC	5000	3.46	1.275	1.80	0.066
MC	6000	3.46	1.267	1.80	0.066
MC	7000	3.49	1.237	1.80	0.071
MC	8000	3.50	1.229	1.80	0.071
MC	9000	3.51	1.222	1.80	0.072
MC	10,000	3.51	1.221	1.80	0.070
V05	1500	4.32	0.408	1.63	0.009
V1	2000	4.40	0.335	1.18	0.011
V2	3000	4.44	0.290	1.34	0.008
V4	5000	4.57	0.156	1.29	0.004
V8	9000	4.56	0.171	1.08	0.005

**Table 6:** Pareto front indicators for different number of surrogate model learning patterns, frequency bands.

Surrogate Model	FE Calls	$I_{H}$↑	$I_{H2}$↓	$I_{\epsilon 1}$↓	$I_{\mathrm{GD}}$↓	Surrogate Model	FE Calls	$I_{H}$↑	$I_{H2}$↓	$I_{\epsilon 1}$↓	$I_{\mathrm{GD}}$↓
50 Hz						60 Hz					
MC	1000	0.28	1.392	2.32	0.395	MC	1000	0.28	0.835	2.22	0.467
MC	2000	0.28	1.392	2.22	0.419	MC	2000	0.28	0.835	2.22	0.354
MC	3000	0.52	1.146	2.22	0.357	MC	3000	0.28	0.835	2.22	0.441
MC	4000	0.52	1.146	2.22	0.476	MC	4000	0.28	0.835	2.22	0.373
MC	5000	0.56	1.105	2.22	0.558	MC	5000	0.28	0.835	2.22	0.321
MC	6000	0.59	1.080	1.86	0.485	MC	6000	0.32	0.795	2.22	0.256
MC	7000	0.59	1.080	1.86	0.485	MC	7000	0.32	0.795	2.22	0.252
MC	8000	0.59	1.080	1.86	0.485	MC	8000	0.32	0.790	2.22	0.228
MC	9000	0.59	1.080	1.86	0.485	MC	9000	0.34	0.779	2.10	0.223
MC	10,000	0.59	1.080	1.86	0.485	MC	10,000	0.34	0.779	2.10	0.223
V05	1493	0.00	1.668	2.84	0.218	V05	1082	0.33	0.786	3.23	1.071
V1	1839	0.70	0.966	1.64	0.074	V1	1226	0.79	0.320	1.74	0.110
V2	2732	1.05	0.613	1.52	0.055	V2	2307	0.91	0.203	1.41	0.097
V4	4802	1.15	0.518	1.25	0.054	V4	4283	1.02	0.091	1.22	0.048
V8	8776	1.12	0.551	1.31	0.058	V8	8394	1.03	0.085	1.41	0.052
70 Hz						80 Hz					
MC	1000	0.78	1.289	2.16	0.485	MC	1000	1.17	2.753	3.38	0.258
MC	2000	0.80	1.269	2.00	0.414	MC	2000	1.65	2.272	2.79	0.249
MC	3000	0.82	1.246	1.84	0.374	MC	3000	1.83	2.097	2.79	0.222
MC	4000	0.82	1.246	1.84	0.374	MC	4000	1.89	2.034	2.79	0.199
MC	5000	0.82	1.243	1.84	0.370	MC	5000	1.90	2.021	2.79	0.193
MC	6000	0.96	1.104	1.84	0.337	MC	6000	1.97	1.954	2.77	0.182
MC	7000	0.99	1.075	1.84	0.276	MC	7000	2.07	1.858	2.77	0.164
MC	8000	1.06	1.010	1.84	0.288	MC	8000	2.07	1.857	2.77	0.155
MC	9000	1.06	1.010	1.84	0.267	MC	9000	2.07	1.855	2.77	0.152
MC	10,000	1.06	1.010	1.84	0.267	MC	10,000	2.15	1.771	2.61	0.148
V05	1397	0.92	1.143	1.95	0.149	V05	1433	2.46	1.464	2.80	0.089
V1	1733	1.66	0.407	1.64	0.070	V1	1946	2.89	1.039	1.35	0.062
V2	2720	1.72	0.349	1.34	0.037	V2	2810	3.49	0.439	1.22	0.028
V4	4848	1.91	0.154	1.26	0.034	V4	4821	3.69	0.235	1.21	0.022
V8	8610	1.94	0.121	1.18	0.018	V8	8801	3.67	0.255	1.20	0.020

**Table 7:** Pareto fronts, highest frequency values.

	V4	V4	TPF	TPF
	$f_{1}$ **or Bandwidth**	**Cost**	$f_{1}$ **or Bandwidth**	**Cost**
	**[Hz]**	**[-]**	**[Hz]**	**[-]**
$f_{1}$	44.55	3.36	46.19	3.55
50 Hz	22.97	4.42	23.04	3.90
60 Hz	23.29	1.11	23.93	1.11
70 Hz	27.32	2.16	28.02	1.71
80 Hz	30.99	1.86	31.64	2.31

## Data Availability

The data underlying this article will be shared on reasonable request from the corresponding author.

## Abbreviations

The following abbreviations are used in this manuscript:

CFRP	Carbon Fiber Reinforced Polymer
DNN	Deep Neural Networks
FEA	Finite Element Analysis
FEM	Finite Element Method
GA	Genetic Algorithm
GFRP	Glass Fiber Reinforced Polymer
MC	Monte Carlo
NSGA-II	Nondominated Sorting Genetic Algorithm II
PSO	Particle Swarm Optimization
*t*FRP	Theoretical Fiber Reinforced Polymer
TPF	True Pareto Front
